# LPA, HGF, and EGF utilize distinct combinations of signaling pathways to promote migration and invasion of MDA-MB-231 breast carcinoma cells

**DOI:** 10.1186/1471-2407-13-501

**Published:** 2013-10-27

**Authors:** Susan MW Harrison, Teresa Knifley, Min Chen, Kathleen L O’Connor

**Affiliations:** 1 Markey Cancer Center, University of Kentucky, 741 S. Limestone Street, Lexington 40506-0509, USA; 2 Department of Molecular and Cellular Biochemistry, University of Kentucky, Lexington 40506-0509, USA; 3 Graduate Center for Toxicology, University of Kentucky, Lexington 40506-0509, USA

**Keywords:** Rho GTPases, RhoA, RhoC, ROCK, Rac1, MAPK, MLCK, Breast carcinoma, Chemotaxis, Invasion

## Abstract

**Background:**

Various pathways impinge on the actin-myosin pathway to facilitate cell migration and invasion including members of the Rho family of small GTPases and MAPK. However, the signaling components that are considered important for these processes vary substantially within the literature with certain pathways being favored. These distinctions in signaling pathways utilized are often attributed to differences in cell type or physiological conditions; however, these attributes have not been systematically assessed.

**Methods:**

To address this question, we analyzed the migration and invasion of MDA-MB-231 breast carcinoma cell line in response to various stimuli including lysophosphatidic acid (LPA), hepatocyte growth factor (HGF) and epidermal growth factor (EGF) and determined the involvement of select signaling pathways that impact myosin light chain phosphorylation.

**Results:**

LPA, a potent stimulator of the Rho-ROCK pathway, surprisingly did not require the Rho-ROCK pathway to stimulate migration but instead utilized Rac and MAPK. In contrast, LPA-stimulated invasion required Rho, Rac, and MAPK. Of these three major pathways, EGF-stimulated MDA-MB-231 migration and invasion required Rho; however, Rac was essential only for invasion and MAPK was dispensable for migration. HGF signaling, interestingly, utilized the same pathways for migration and invasion, requiring Rho but not Rac signaling. Notably, the dependency of HGF-stimulated migration and invasion as well as EGF-stimulated invasion on MAPK was subject to the inhibitors used. As expected, myosin light chain kinase (MLCK), a convergence point for MAPK and Rho family GTPase signaling, was required for all six conditions.

**Conclusions:**

These observations suggest that, while multiple signaling pathways contribute to cancer cell motility, not all pathways operate under all conditions. Thus, our study highlights the plasticity of cancer cells to adapt to multiple migratory cues.

## Background

The motility of a cell is determined by its ability to coordinately regulate a dynamic organization of the cytoskeletal architecture to create polarity, rigidity and contractile forces necessary for movement. At the core of a cell’s ability to migrate is the interaction between actin and non-muscle myosin II, whose activation states cycle in a systematically regulated manner (reviewed in [1,2]). Actin polymerizes and depolymerizes on a continuous basis and typically forms a meshwork with protrusions at the leading edge of the cell, pushing the plasma membrane forward. At the rear of the cell, long, unbranched actin-myosin filaments mediate contraction that pulls the rear of the cell forward and retraction of the trailing edge to facilitate cell polarization that promotes directed cell migration. During these coordinated processes, myosin II and its regulatory myosin light chain (MLC) undergo cycles of phosphorylation and dephosphorylation; these changes affect the conformational state of myosin, allow it to interact with actin, and move actin fibers relative to each other. In concert with the directed polymerization of actin, the motor activity of myosin results in cell propulsion.

**Figure 1:**
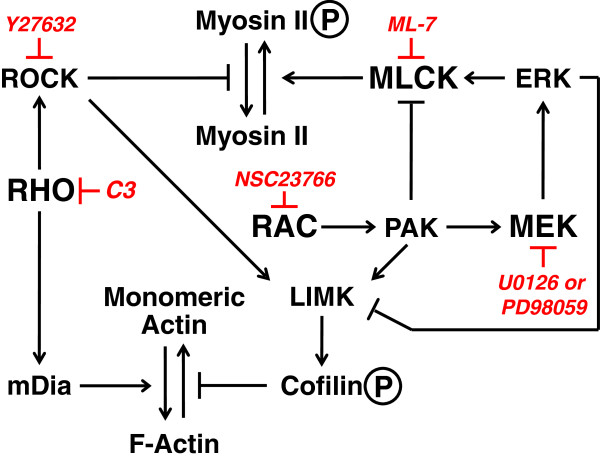
**Signaling pathways important for the control of actin dynamics and myosin activity.** Key signaling pathways that converge on actin and myosin to control cell motility are depicted and include Rho, Rac, MLCK, MAPK/MEK. The inhibitors for each of these pathways used in this study are indicated in italics.

Numerous well-studied signal transduction pathways converge to control the activity of actin and myosin II (Figure 1) and, hence, cytoskeletal architecture and movement. Among the most influential are the Rho GTPases, Rho and Rac (reviewed in [3,4]) but also include the MEK/Erk mitogen-activated protein kinase (MAPK) pathway [5]. Rho promotes both actin polymerization and myosin II contractility. Rho-induced actin polymerization is mediated by the Rho effector mammalian homologue of diaphanous (mDia) [6], a member of the formin family, while myosin II activity is promoted through Rho-associated coiled coil kinase (ROCK) control of myosin by inhibiting myosin phosphatase [7,8]. Preventing myosin phosphatase from dephosphorylating MLC prolongs MLC and thus myosin activity. Rac, on the other hand, can inhibit myosin light chain kinase (MLCK) to reduce myosin II activity while simultaneously promoting actin polymerization; both of these functions can be attributed to p21-activated kinases (PAK) [9,10]. Recently, PAK was shown to be central to the flow of actin in the lamella and the localization of myosin II at the leading edge to facilitate cell migration [11]. The Rho and Rac pathways converge on LIM-kinase (LIMK) downstream of ROCK [12] and PAK [13], respectively, which leads to the phosphorylation and inactivation of the F-actin depolymerizing protein cofilin, thereby stabilizing actin filaments [14]. MAPK has been shown to limit activation of LIMK, subsequent phosphorylation of cofilin, and migration of primary human T cells in a three-dimensional (3D) environment [15]. The MAPK cascade also regulates myosin II activity by phosphorylating and enhancing the activity of MLCK [16]. Therefore, control of actomyosin dynamics results from cooperation of multiple signaling pathways that have independent effects on both actin and myosin which must be balanced appropriately to allow cell movement.

The pathways presented above, however, have been pieced together from data obtained using a wide variety of cell types subjected to a myriad of conditions and all have been implicated in one way or another with metastasis of carcinoma cells. However, the signaling components that are considered important for these processes vary substantially in the literature with certain pathways being favored. One could effectively argue that distinctions in the signaling pathways utilized are a result of variations in cell types or handling by investigators. However, they might also arise from differences in physiological conditions, which have not been systematically evaluated.

Here, we dissect the pathways used by highly metastatic breast carcinoma cell line MDA-MB-231, which are able to migrate and invade toward LPA, HGF and EGF [17-19]. We find that partially overlapping subsets of signaling pathways are employed by these cells depending on the environmental context. This observation has important consequences for cancer therapy and rational drug design.

## Methods

### Cells treatments and reagents

The metastatic MDA-MB-231 breast carcinoma cell line was cultured in low glucose DMEM (Invitrogen), 10% Fetalplex (Gemini Bio-Products, West Sacramento CA), 1% penicillin-streptomycin, 1% l-glutamine (Invitrogen) to 70% confluence. For experiments, cells were trypsinized and rinsed with DMEM plus 250 μg/ml fatty acid-free BSA (Gemini Bio-Products cat# 700-107P) (DMEM/BSA). For pharmacological inhibitor studies, cells were treated in suspension for 30 min at 37°C with the following inhibitors: 10 μM ML-7 (MLCK inhibitor, Calbiochem cat# 475880), 10 μM U0126 (MEK1/2 inhibitor, Calbiochem cat# 662005), 50 μM PD98059 (MEK1/2 inhibitor, Calbiochem cat# 513000), 10 μM Y-27632 (ROCK inhibitor Cayman Chemical cat# 10005583), or 100 μM NSC23766 (Rac inhibitor, Tocris Bioscience cat#2161). To inhibit Rho directly with C3 exotransferase, cells (3×10^6^) were electroporated in serum-free DMEM with 5 μg glutathione-S-transferase (GST; control) or GST-C3 purified bacterial-expressed protein as done previously [20,21] just prior to use.

### Cell migration and invasion assays

For migration assays, transwell chambers were coated with 15 μg/ml collagen I (BD Biosciences cat# 354249) as previously described [18]. For invasion assays, transwells were top-coated with 5 μg Matrigel (BD Biosciences cat# 354234), dried overnight, and rehydrated in 50 μl DMEM for 30 min at 37°C prior to assay; bottom wells were coated with 15 μg/ml collagen I. Chemotaxis or chemoinvasion was stimulated by adding to the bottom chamber 100 nM oleoyl-L-alpha-lysophosphatidic acid sodium salt (LPA, Sigma cat# L-7269), 50 ng/ml HGF (PeproTech cat# 100–39) or 5 ng/ml EGF (PeproTech cat# AF-100-15) diluted in DMEM/BSA, in the absence or presence of inhibitor. Cells (5×10^4^) were allowed to migrate or invade at 37°C for 3 hours in the presence of inhibitors, as noted. Data are presented as the mean cell number migrated per mm^2^ from triplicate wells and plotted with the standard deviation. Statistical significance was calculated using a two-tailed unpaired t-test assuming equal variances. For all experiments, data presented are representative of a minimum of three independent assays. In some cases, controls (cells not treated with chemoattractant or inhibitor) are reported more than once in a figure if multiple conditions for the same experiment were represented for consistency of the presentation.

### MAPK, Rho and Rac activity assays

Cells were plated onto collagen-coated dishes for 3 hrs. Cells were then treated with 10 μM U0126 or 50 μM PD98059 before stimulation with 100 nM LPA, 50 ng/ml HGF or 5 ng/ml EGF. The activity of the MAPK pathway was determined by immunoblot analysis of p44/p42 MAPK (Erk 1/Erk2) for total Erk (3A7, mouse Ab, Cell Signaling) and the phosphorylated form (197G2, rabbit Ab, Cell Signaling).

Rho assays were assessed using a Rhotekin RBD affinity assay as described previously [18,21,22]. For Rac assays, cells were serum starved overnight, treated with or without 100 μM NSC23766 compound for 3 hrs and then treated with chemoattractant as noted for 5 minutes prior to harvesting. Cell lysates were then assessed for Rac activity using a Pak1 Rac Binding Domain assay, as we have performed previously [21,23].

## Results

### MLCK is involved in migration and invasion of MDA-MB-231 cells

**Figure 2:**
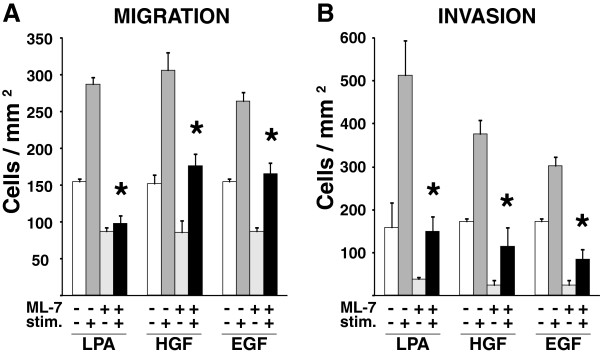
**MLCK is required for cell migration and invasion in response to LPA, HGF and EGF.** MDA-MB-231 cells were allowed to migrate across a collagen-coated transwell **(A)** or invade through Matrigel **(B)** toward LPA, HGF or EGF for 3 hours in the presence or absence of the MLCK inhibitor, ML-7 (inhib.), as noted. The numbers of cells migrated or invaded per mm^2^ were graphed with their standard deviations for triplicate wells within a single experiment. For simplicity of presentation, statistically significant differences between stimulated and stimulated plus inhibitor-treated cell conditions only were determined using a t-test (*p < 0.02). Data are representative of a minimum of three independent assays for each condition.

For these studies, we utilized chemical inhibitors of select signaling molecules (Figure 1) and performed short-term (3 hrs) migration and invasion assays. This approach permits assessment of the immediate downstream impact of select signaling pathways without substantially altering transcription or proliferation that could affect our interpretation. Myosin activation by phosphorylation of the MLC is essential for its interaction with actin and actin-myosin contractility. MLCK is the primary kinase that controls MLC phosphorylation. To determine if MLCK plays a central role in the migration and invasion of MDA-MB-231 cells, we performed transwell migration assays and Matrigel invasion assays using LPA, HGF, or EGF as chemoattractants in the presence of the MLCK inhibitor ML-7. As shown in Figure 2, there was a significant decrease in the number of ML-7 treated cells that migrated or invaded compared to untreated cells, including both basal and growth factor stimulated conditions. The effect was stronger on invasion than migration with a greater than 50% reduction in number of cells that were able to invade in the presence of inhibitor. Therefore, these data demonstrate that MLCK is a key mediator of both migration and invasion in response to a variety of chemotactic agents for MDA-MB-231 cells.

### MAPK pathway in migration and invasion of MDA-MB-231 cells

**Figure 3:**
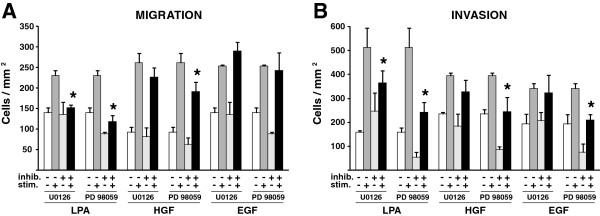
**MAPK pathway has distinct effects on migration and invasion in response to LPA, HGF and EGF.** MDA-MB-231 cells were treated with one of two MEK1/2 inhibitors, U0126 or PD98059, and then assessed for either migration **(A)** or invasion **(B)** toward the indicated chemoattractant. Cells migrated or invaded were then quantified, and results graphed as described in Figure 2. Data are representative of a minimum of three independent assays for each condition. Statistically significant differences between stimulated and stimulated plus inhibitor-treated cell conditions only were determined using a t-test (*p < 0.02).

MAPK directly phosphorylates and activates MLCK, thus facilitating the activation of myosin II *in vitro*[16]. To determine if MAPK influences chemotactic migration or invasion of MDA-MB-231 cells, we used two inhibitors of MEK1 and MEK2, U0126 and PD98059, in migration and invasion assays. We found inhibitor- and chemoattractant–specific effects for both migration and invasion. Both inhibitors significantly reduced the number of cells that migrated or invaded toward LPA (Figure 3). When HGF was used to stimulate cells, U0126 did not affect either migration or invasion while the PD98059 inhibitor reduced both; however, there was a trend toward inhibition with the U0126. Neither inhibitor reduced migration toward EGF while PD98059 was effective in reducing invasion of these cells. The concentration of both of the inhibitors used here were equally effective at reducing ERK phosphorylation, which reflects the MEK1/2 activity, in these cells as analyzed by immunoblot (Additional file 1: Figure S1). Further experiments were performed using 50 nM PD901, a more potent and specific MEK inhibitor. These experiments gave the same results as the PD98059 compound for cell migration and for U0126 for invasion assays (data not shown). These data suggest that the MAPK pathway acts to finely tune migration and invasion responses to different stimuli, but is not required under all conditions.

### Rac and Rho GTPase pathways in migration and invasion of MDA-MB-231 cells

**Figure 4:**
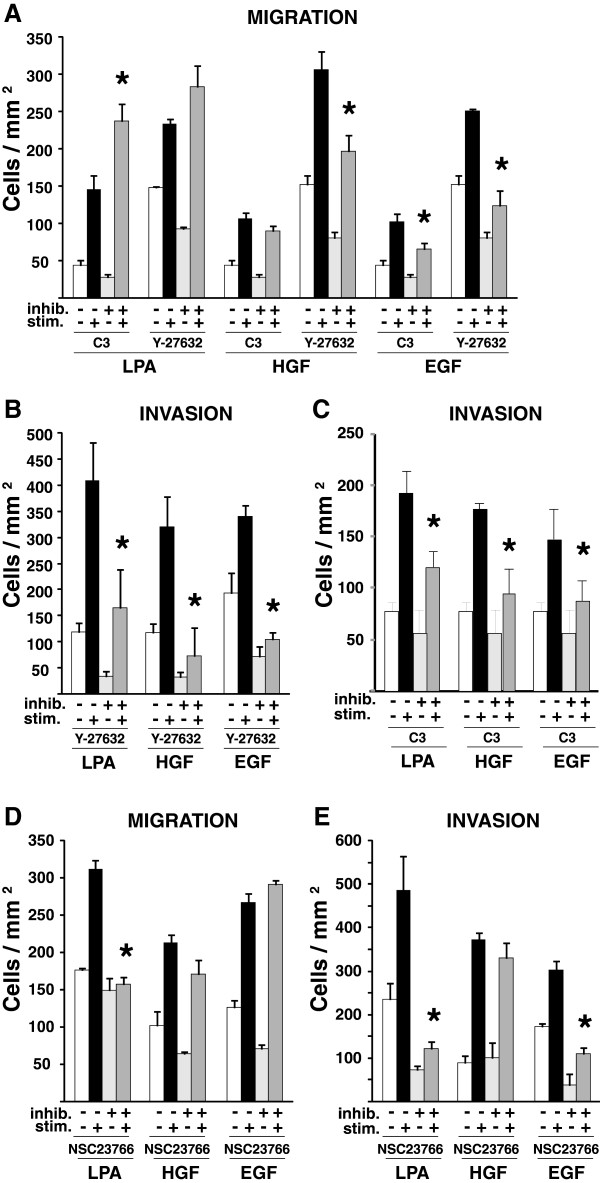
**Rac and Rho GTPase inhibitors have differential effects on migration and invasion in response to LPA, HGF and EGF.** MDA-MB-231 cells were treated with inhibitors of the Rho pathway using C3 or Y-27632 **(A, B, C)**, or the Rac pathway with NSC23766 **(D, E)** as described in the Methods section, and assayed for their ability to migrate **(A, D)** or invade **(B, C, E)** toward various growth factors as described in Figure 2. Data are representative of a minimum of three independent assays for each condition. Statistically significant differences between stimulated and stimulated plus inhibitor-treated cell conditions only were determined using a t-test (*p < 0.02).

Rac and Rho are both major mediators of cell migration and invasion, and are thought to have distinct functions in the migration and invasion of carcinoma cells [3,4]. Here, we utilized electroporation of C3 protein (which ribosylates and inhibits RhoA, B and C; effectiveness shown in Additional file 2: Figure S2) and the ROCK inhibitor Y27632 to assess the Rho-ROCK pathway; Rac activation was inhibited by NSC23766 [24]. We find that MDA-MB-231 cells had opposing requirements for the Rho and Rac pathways for cell migration. LPA–stimulated cells used Rac but not Rho for migration (Figure 4). In fact, inhibition of Rho with C3 exotransferase and inhibition of Rho’s downstream effector ROCK with Y-27632 tended to increase migration (Figure 4A) suggesting that the Rho pathway normally serves to limit this process in these cells. In accordance with this concept, inhibition of the Rho-ROCK pathway tended to increase membrane ruffling and lamellae formation in response to LPA (data not shown). Notably, Rho/Rock pathway is essential to invasion in all conditions (Figure 4B and C) suggesting that Rho is important mediator of tumor cell invasion. HGF and EGF, in contrast, employ Rho but not Rac for migration. During invasion, however, both Rho and Rac pathways were necessary for EGF, while HGF required only Rho. These data are summarized in Tables 1 and 2. Notably, the Rac inhibitor, NSC23766 effectively blocked Rac activation in response to all three chemoattractants (Additional file 3: Figure S3). Therefore, these observations demonstrate that the chemoattractant and migration/invasion condition dictate which GTPases are utilized.

**Table 1:** Summary of pathways used for growth factor-stimulated migration

	**MLCK**	**MAPK**		**Rho**	**Rac**
**Stimulus**		**(U0126)**	**(PD98059)**		
LPA	YES	YES	YES	NO	YES
HGF	YES	NO	YES	YES	NO
EGF	YES	NO	NO	YES	NO

**Table 2:** Summary of pathways used for growth factor-stimulated invasion

	**MLCK**	**MAPK**		**Rho**	**Rac**
**Stimulus**		**(U0126)**	**(PD98059)**		
LPA	YES	YES	YES	YES	YES
HGF	YES	NO*	YES	YES	NO
EGF	YES	NO	YES	YES	YES

*A trend toward inhibition is noted but does not reach statistical significance.

## Discussion and conclusions

The metastatic process requires cells to leave their native site in response to environmental cues and move to a different location where they proliferate to form new tumors. The cellular mechanisms used in this aberrant migratory response are fundamentally similar to those used during normal developmental migration and boil down to the ability of the cell to organize its actin cytoskeleton in a polarized manner and to activate the myosin motor function to move that cytoskeleton in the direction of polarization [25]. These basic functions can be mediated by a variety of apparently redundant signaling pathways that converge on the polymerization of actin and the activation state of the myosin II. However, we have shown here that in a single cell type context specific motility can signal through different subsets of pathways, as summarized in Tables 1 and 2, despite the fact that they all converge on the activity of a relatively small number of proteins (see Figure 1). Not surprisingly, MLCK is an essential player in both migration and invasion in response to all of the stimuli tested here, as it is central to the control of myosin II phosphorylation and subsequent activation. Beyond that, while capable of employing several pathways to drive actomyosin-mediated movement, these cells utilize specific subsets of pathways to achieve motility in different environments and toward distinct growth factors. These observations demonstrate a surprising versatility and plasticity in carcinoma migration and invasion.

In development and leukocyte navigation, it is well accepted that different chemoattractants will stimulate specific pathways such that patterning and precise navigation can be achieved [26]. Certainly if all cells responded to a stimulus in a similar manner, it would not be conducive to precise navigation or organismal development. However, the concept that tumor cells might utilize multiple growth factor signaling pathways for dissemination in different ways is not generally discussed. Rather the responses to specific growth factors or cocktails of factors (such as fibroblast conditioned medium) are reported in such a way that these conditions likely apply to all cancer cells, or at least a particular subtype of cancer. Furthermore, modes of migration can vary substantially among tumor cells [25,27]. Considerable attention has been given to the role of the extracellular environment, its matrix composition, and matrix tension in mediating the differences in cancer cell invasion. However, how select signaling events from distinct growth factors and other chemoattractants might facilitate these differences has received noticeably less scrutiny. One exception is the roles of Rac and Rho pathways. Rac is generally attributed to the formation of lamellae, which are used to propel cells forward. These large fan-shaped lamellae are advantageous for two-dimensional migration but may in fact be inhibitory for movement through a 3D environment where space is at a premium, especially when matrix metalloprotease activity is low [27]. In the 3D condition, a smaller pseudopodial-like protrusion might be more advantageous. Importantly, RhoA has also been implicated in membrane ruffling and lamellae formation [6,22,28] where it can play a major role in 3D invasion [7,17,29,30]. Notably, the utilization of RhoA in the formation of lamellae can be in cooperation with [21] or independent from Rac1 [22]. The types of protrusions formed by Rac, Rho or the cooperation of Rac and Rho are expected to be functionally redundant, but could in fact be fundamentally different in structure or their function altered by the 3D environment. Certainly more research is needed in this regard with careful attention to the fact that the role of these GTPases in invasion may be multifaceted.

We further find that the MAPK pathway is an important mediator of LPA chemotaxis and invasion, but is dispensable for EGF-mediated migration. On the other hand, the MAPK pathway plays a less definitive role in the migration and invasion of these cells toward HGF. The inconsistences observed in the migration and invasion behavior of MDA-MB-231 cells in response to different inhibitors of MEK, specifically in invasion toward EGF and migration and invasion toward HGF, raises some question as to how the MAPK pathway contributes to these events. While PD98059 and U0126 are MEK1/2 inhibitors, these two inhibitors work by distinct mechanisms. Furthermore, U0126 inhibits both MEK1 and MEK2 while PD98059 has a more potent effect on MEK1 than on MEK2 [31,32]. The discrepancy in the results using the two inhibitors could indicate differences in utilization of MEK1 versus MEK2, or that simply off target effects of the inhibitors alter the interpretation. Previous studies have noted similar differences between the two compounds where PD98059 inhibits preferentially [33], thus suggesting MEK2 might counteract the function of MEK1. Certainly more definitive experiments will be required to fully elucidate the role of the MAPK pathway, including substrate specificity and individual contributions of each kinase.

This study was not meant to be a comprehensive analysis of signaling pathways in response to the conditions assessed here. But rather we sought to demonstrate that migration of carcinoma cells, even a single cell line, is more versatile than previously recognized. Here, we chose to use pathway specific inhibitors and short term assays rather than genetic analyses to distinguish the immediate signaling effects of these pathways from the effects on transcription or proliferation that might alter our interpretation of the results. Accordingly, our results do not conclude, as an example, that Pak is unimportant to select migration or invasion conditions where the Rac inhibitor shows no effect. Pak could be stimulated by cdc42 or Rac3, which are not reported targets of the Rac inhibitor [24]. Notably, transcriptomes vary from cell to cell. And with these variations come differences in how pathways are activated, spatially localized, and utilized during specific signaling contexts. Since we use a single cell line for this study, we find that a cell can use specific combinations of pathways to achieve migration or invasion in response to different stimuli that goes beyond cell-to-cell variations in transcriptional profiles. The use of some but not all of the possible pathways to control actin reorganization has been seen in other cell types in response to particular environments. Clone A colon carcinoma cells use MAPK signaling (unpublished observation) and Rho, but not Rac, to drive migration on laminin [22]. In contrast, MDA-MB-435 cells use Rac [23] and Rho [21] but not MAPK (unpublished observation) to migrate toward LPA, which differs from the MDA-MB-231 cells.

To determine which signaling pathways govern the motility and invasion of a particular cell type, the physiological conditions including matrix composition, matrix compliance, and growth factors utilized to stimulate these processes need to be considered. This concept becomes particularly important when screening genes and compounds for their impact on tumor invasion. As an example, fetal bovine serum (FBS) is a common stimulant for migration and invasion assays. The major pro-migratory growth factor in FBS is LPA, which is found in concentrations as high as 20 μM [34]. We have found previously that these high levels of LPA (1 μM and higher) can actually inhibit the migration of breast carcinoma cells [18]. In this study, we find that LPA does not require Rho signaling for chemotaxis, but does for invasion. Therefore, the use of FBS in migration or invasion would have low potential to yield important insight into the breast cancer invasion process. Furthermore, if screens utilize FBS in migration assays to represent the tumor invasion process, important compounds might be discarded in the *in vitro* screening process, thus eliminating potentially effective drugs in lieu of ones that ultimately may be ineffective *in vivo*. Consequently, our study helps to highlight the importance of physiological context when assessing pertinent signal transduction pathways, which has notable implications for the effective development of cancer therapeutics and rational drug design.

## Abbreviations

EGF: Epidermal growth factor; FBS: Fetal bovine serum; GST: Glutathione-S-transferase; HGF: Hepatocyte growth factor; LPA: Lysophosphatitic acid; mDia: Mammalian homologue of diaphanous; MAPK: Mitogen-activated protein kinase; MLC: Myosin light chain; MLCK: Myosin light chain kinase; PAK: P21-activated kinases; ROCK: Rho-associated coiled coil kinase; 3D: Three-dimensional.

## Competing interests

The authors declare that they have no competing interests.

## Authors’ contributions

SMWH and KLO designed and wrote up the current study. MC was involved in the study design, validation of Rho and Rac inhibition, and editing the manuscript. TK and SMWH performed all experiments and analyzed all data. All authors read and approved the final manuscript.

## Pre-publication history

The pre-publication history for this paper can be accessed here:

http://www.biomedcentral.com/1471-2407/13/501/prepub

## Supplementary Material

Additional file 1: Figure S1 The Mek inhibitors U0126 and PD98059. MDA-MB-231 cells were plated onto collagen coated dishes for 3 hrs and then either left untreated or treated with 10 μM U0126 or 50 μM PD98059 30 mins, as noted. Cells were then stimulated with 100 nM LPA, 50 ng/ml HGF, or 5 ng/ml EGF for 5 mins before lysis. Cell lysates were then analyzed for phospho p44/p42 MAPK (Erk 1/2; upper bands) and total p44/p42 MAPK by immunoblot analysis. Click here for file

Additional file 2: Figure S2 Confirmation of C3 treatment on RhoA inhibition. MDA-MB-231 cells were electroporated with bacterially expressed GST or GST-C3, plated on collagen coated plates for 2 hrs and then stimulated with 100 nM LPA for 5 mins. Cell lysates were then analyzed for RhoA activity using a GST-Rhotekin RBD binding assay (upper bands; active) and total RhoA (10% total; bottom bands) by immunoblot analysis. Click here for file

Additional file 3: Figure S3 NSC23766 effectively inhibits Rac in response to LPA, HGF or EGF stimulation. MDA-MB-231 cells were serum starved overnight and then treated with 100 μM of the Rac inhibitor NSC23766 for 3 hours, as noted. Cell were then stimulated with BSA (control), 100 nM LPA, 50 ng/ml HGF, or 5 ng/ml EGF for 5 mins. before lysis. Cell lysates were then analyzed for Rac activity using a GST-Pak RBD binding assay (upper bands; active) and total Rac (10% total; bottom bands) by immunoblot analysis. Numbers below the blots represent fold activity compared to untreated control cells. Click here for file
