# Supplementary figures and images for: LPA, HGF, and EGF utilize distinct combinations of signaling pathways to promote migration and invasion of MDA-MB-231 breast carcinoma cells
Source: BMC Cancer. 2013 Oct 27;13:501. doi: 10.1186/1471-2407-13-501 (PMC3819718; doi:10.1186/1471-2407-13-501)

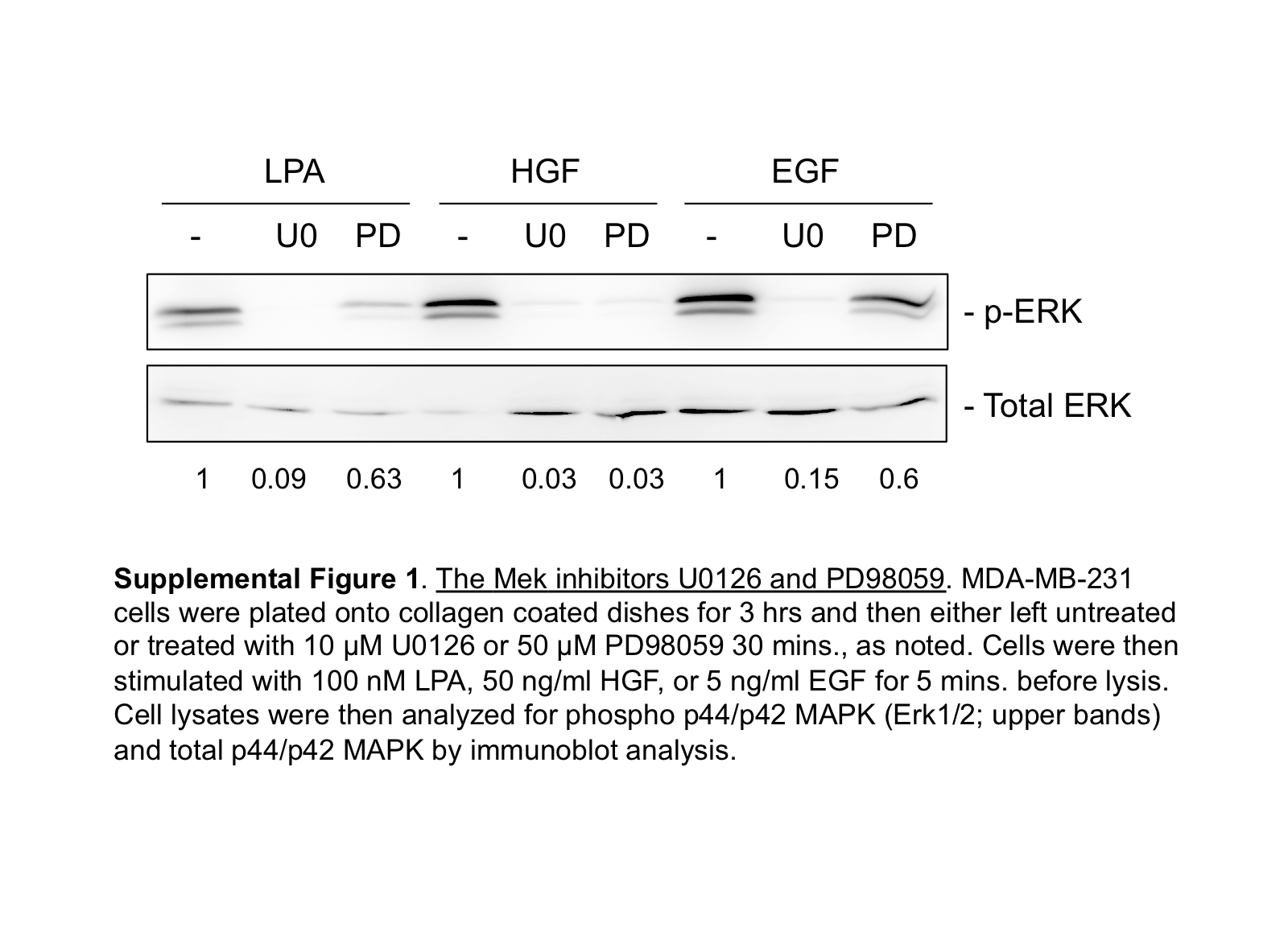

Supplement: Additional file 1: Figure S1 — The Mek inhibitors U0126 and PD98059. MDA-MB-231 cells were plated onto collagen coated dishes for 3 hrs and then either left untreated or treated with 10 μM U0126 or 50 μM PD98059 30 mins, as noted. Cells were then stimulated with 100 nM LPA, 50 ng/ml HGF, or 5 ng/ml EGF for 5 mins before lysis. Cell lysates were then analyzed for phospho p44/p42 MAPK (Erk 1/2; upper bands) and total p44/p42 MAPK by immunoblot analysis. [file 1471-2407-13-501-S1.tiff]

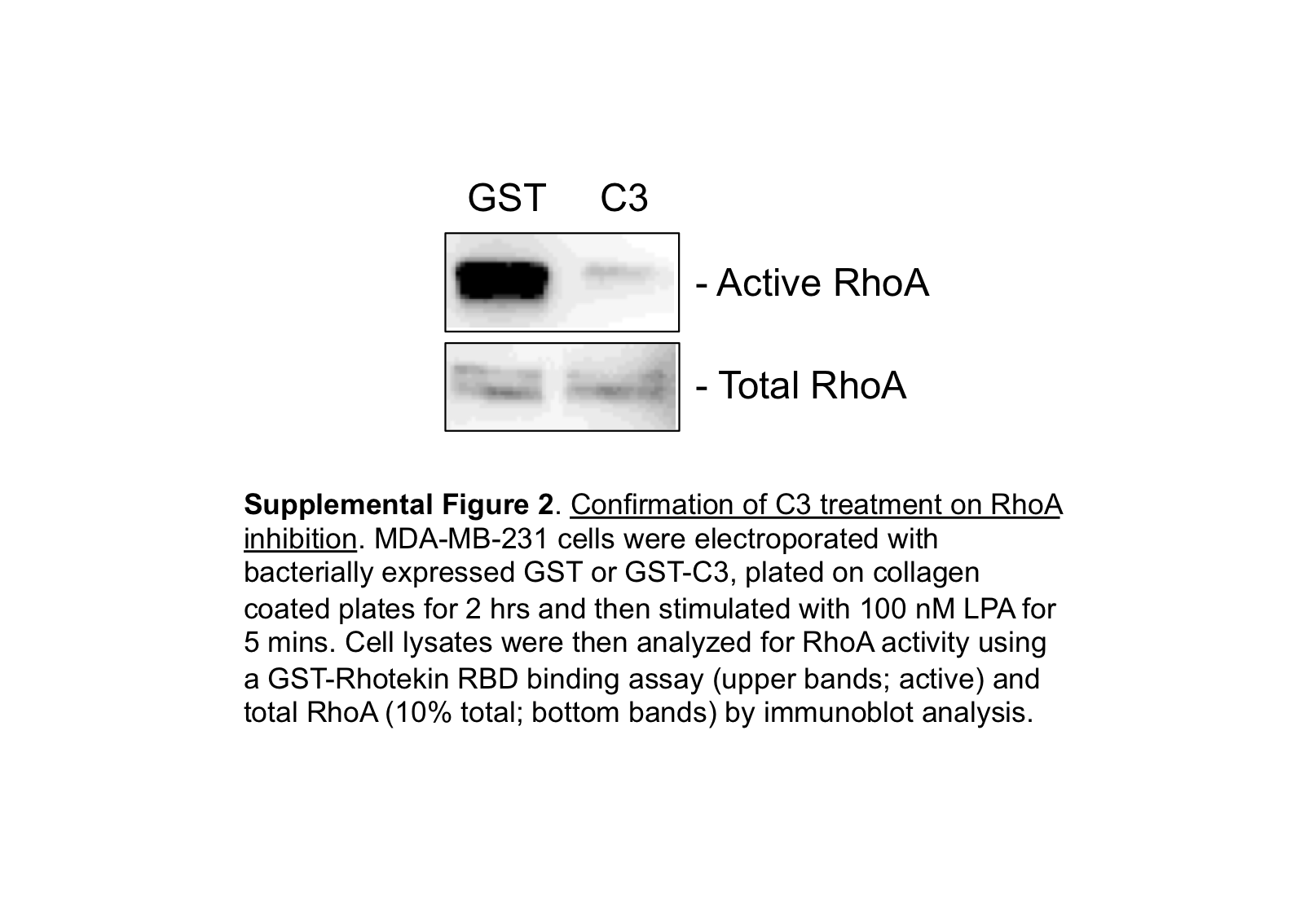

Supplement: Additional file 2: Figure S2 — Confirmation of C3 treatment on RhoA inhibition. MDA-MB-231 cells were electroporated with bacterially expressed GST or GST-C3, plated on collagen coated plates for 2 hrs and then stimulated with 100 nM LPA for 5 mins. Cell lysates were then analyzed for RhoA activity using a GST-Rhotekin RBD binding assay (upper bands; active) and total RhoA (10% total; bottom bands) by immunoblot analysis. [file 1471-2407-13-501-S2.tiff]

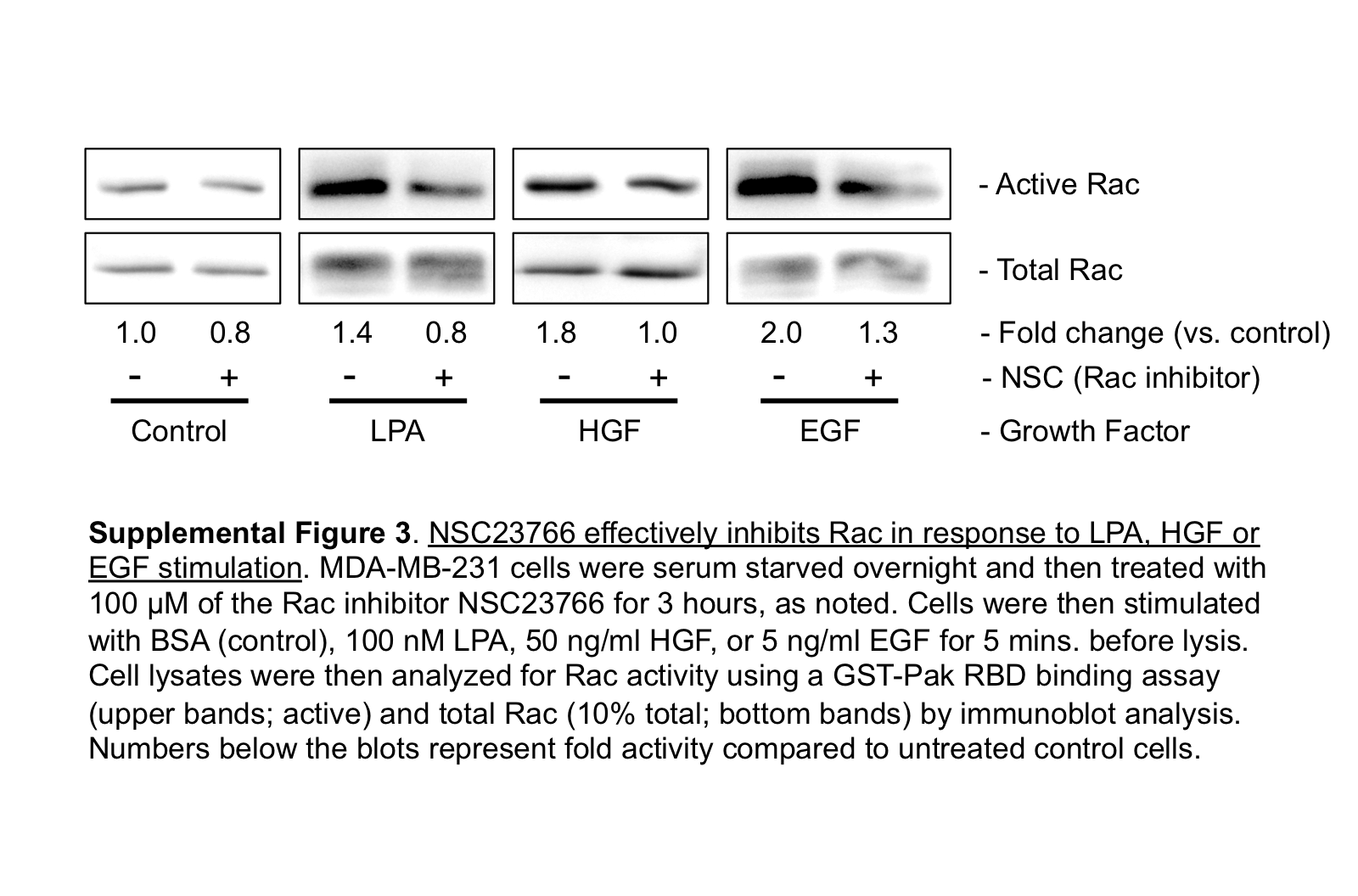

Supplement: Additional file 3: Figure S3 — NSC23766 effectively inhibits Rac in response to LPA, HGF or EGF stimulation. MDA-MB-231 cells were serum starved overnight and then treated with 100 μM of the Rac inhibitor NSC23766 for 3 hours, as noted. Cell were then stimulated with BSA (control), 100 nM LPA, 50 ng/ml HGF, or 5 ng/ml EGF for 5 mins. before lysis. Cell lysates were then analyzed for Rac activity using a GST-Pak RBD binding assay (upper bands; active) and total Rac (10% total; bottom bands) by immunoblot analysis. Numbers below the blots represent fold activity compared to untreated control cells. [file 1471-2407-13-501-S3.tiff]
